# Framework for Multistakeholder Patient Registries in the Field of Rare Diseases: Focus on Neurogenetic Diseases

**DOI:** 10.1212/WNL.0000000000209743

**Published:** 2024-08-22

**Authors:** Daphne H. Schoenmakers, Sibren van den Berg, Lonneke Timmers, Laura A. Adang, Tobias Bäumer, Annet Bosch, Marc van de Casteele, Mareen R. Datema, Hanka Dekker, Conan Donnelly, Mariëtte H.E. Driessens, Holm Graessner, Valerie Greger, Tala Haddad, Günter U. Höglinger, Hannerieke van den Hout, Carla Jonker, Mirjam Langeveld, Laurie J. Lambert, Eileen Neacy, Marc Nieuwland, Thomas Klockgether, Marjo S. van der Knaap, Andri Papadopoulou, Kelly Plueschke, Sanne van Rijn, Noa Rosenberg, Elise F. Saunier-Vivar, Bruna dos Santos Vieira, Carla E.M. Hollak, Wim G. Goettsch, Nicole I. Wolf

**Affiliations:** From the Department of Child Neurology (D.H.S., M.S.v.d.K., N.I.W.), Emma's Children's Hospital, Amsterdam UMC location Vrije Universiteit; Amsterdam Leukodystrophy Center (D.H.S., M.S.v.d.K., N.I.W.), Amsterdam Neuroscience, Cellular & Molecular Mechanisms; Medicine for Society (D.H.S., S.v.d.B., N.R., C.E.M.H.), Platform at Amsterdam UMC location University of Amsterdam; Department of Endocrinology and Metabolism (S.v.d.B., A.B., M.R.D., N.R., C.E.M.H.), Amsterdam UMC location University of Amsterdam; National Health Care Institute (Zorginstituut Nederland) (L.T.), Diemen, the Netherlands; Division of Child Neurology (L.A.A.), Children's Hospital of Philadelphia, PA; Institute of Systems Motor Science (T.B.), CBBM, Universität of Lübeck; Centre of Rare Diseases (T.B.), University Hospital Schleswig Holstein, Lübeck, Germany; Division of Metabolic Diseases (A.B.), Department of Pediatrics, Emma Childrens' Hospital, Amsterdam UMC location University of Amsterdam, the Netherlands; National Health Care Institute RIZIV-INAMI (M.v.d.C.), Brussels, Belgium; VKS (H.D.), Dutch Patient Organization for Metabolic Diseases, Zwolle; United for Metabolic Diseases (UMD) (H.D.), Amsterdam, the Netherlands; International Niemann-Pick Disease Registry (C.D.), Washington, Tyne & Wear, United Kingdom; VSOP-Patient Alliance for Rare and Genetic Diseases (M.H.E.D.), Soest, the Netherlands; Institute for Medical Genetics and Applied Genomics (H.G.), University of Tübingen; Centre for Rare Disease (H.G.), University Hospital Tübingen, Germany; Yaya foundation for 4H Leukodystrophy (V.G.), Minneapolis, MN; Orphanet (T.H.), INSERM US14 Rare Disease Platform, Paris, France; Department of Neurology (G.U.H.), LMU University Hospital, Ludwig-Maximilians-Universität (LMU), Munich; German Center for Neurodegenerative Diseases e.V. (DZNE) (G.U.H., T.K.), Munich; Munich Cluster for Systems Neurology (SyNergy) (G.U.H.), Germany; Department of Pediatrics (H.v.d.H.), Center for Lysosomal and Metabolic Diseases, Erasmus MC University Medical Center, Sophia Children's Hospital, Rotterdam; European Medicines Agency (C.J., K.P.), Amsterdam; Medicines Evaluation Board (C.J.), Utrecht; Department of Endocrinology and Metabolism (M.L.), Amsterdam UMC, Amsterdam Gastroenterology Endocrinology Metabolism (AGEM) Research Institute, University of Amsterdam, the Netherlands; Canadian Agency for Drugs and Health Technology Technologies Agendcy in Health (CADTH) (L.J.L.), Ottawa, Ontario, Canada; CHDI Management, Inc. (E.N.), the company that manages the scientific activities of CHDI Foundation, Inc., New York, NY; National Health Care Institute (M.N., W.G.G.), Diemen, the Netherlands; Department of Neurology (T.K.), University of Bonn, Germany; Department of Integrative Neurophysiology (M.S.v.d.K.), Center for Neurogenomics and Cognitive Research, Vrije Universiteit, Amsterdam, the Netherlands; European Commission (A.P.), Joint Research Centre (JRC), Ispra, Italy; Patient Advocate Organization ‘Vereniging HCHWA-d’ (HCHWA-D Association) (S.v.R.), the Netherlands; European Leukodystrophies Association (E.F.S.-V.), Paris, France; Medical BioSciences Department (B.d.S.V.), Radboud University Medical Center, Nijmegen; and WHO Collaborating Centre for Pharmaceutical Policy and Regulation (W.G.G.), Division of Pharmacoepidemiology and Clinical Pharmacology, Utrecht University, the Netherlands.

## Abstract

Progress in genetic diagnosis and orphan drug legislation has opened doors to new therapies in rare neurogenetic diseases (RNDs). Innovative therapies such as gene therapy can improve patients' quality of life but come with academic, regulatory, and financial challenges. Registries can play a pivotal role in generating evidence to tackle these, but their development requires multidisciplinary knowledge and expertise. This study aims to develop a practical framework for creating and implementing patient registries addressing common challenges and maximizing their impact on care, research, drug development, and regulatory decision making with a focus on RNDs. A comprehensive 3-step literature and qualitative research approach was used to develop the framework. A qualitative systematic literature review was conducted, extracting guidance and practices leading to the draft framework. Subsequently, we interviewed representatives of 5 established international RND registries to add learnings from hands-on experiences to the framework. Expert input on the draft framework was sought in digital multistakeholder focus groups to refine the framework. The literature search; interviews with 5 registries; and focus groups with patient representatives (n = 4), clinicians (n = 6), regulators, health technology assessment (HTA) bodies and payers (n = 7), industry representatives (n = 7), and data/information technology (IT) specialists (n = 5) informed development of the framework. It covers the interests of different stakeholders, purposes for data utilization, data aspects, IT infrastructure, governance, and financing of rare disease registries. Key principles include that data should be rapidly accessible, independent, and trustworthy. Governance should involve multiple stakeholders. In addition, data should be highly descriptive, machine-readable, and accessible through a shared infrastructure and not spread over multiple isolated repositories. Sustainable and independent financing of registries is deemed important but remains challenging because of a lack of widely supported funding models. The proposed framework will guide stakeholders in establishing or improving rare disease registries that fulfill requirements of academics and patients as well as regulators, HTA bodies, and commercial parties. There is a need for more clarity regarding quality requirements for registries in regulatory and HTA context. In addition, independent financing models for registries should be developed, as well as well-defined policies on technical uniformity in health data.

## Introduction

Rare neurogenetic diseases (RNDs) consist of a diverse group of diseases profoundly affecting motor and cognitive function and life expectancy.^1^ While treatments remain limited, advances in genetic diagnosis and orphan drug development and legislations are encouraging. In fact, orphan drugs for RND and inborn errors of metabolism dominate nononcological orphan drugs in both the United States^2-4^ and the European Union.^5^ New therapies such as gene therapy are promising but also pose new academic, regulatory, and financial challenges. These challenges include lack of evidence on (long-term) safety, effectiveness, and appropriate use at the time of market entry and concerns regarding cost-effectiveness and budget impact.

Patient registries are considered to be crucial for development and regulation of orphan drugs for the treatment of RND.^6^ Unlike natural history studies that gather detailed data from untreated patients in controlled settings, registries are systems designed to broadly collect, store, and use real-world data. Registries can aid in increasing trial readiness^1^ and inform trial design and execution.^7-9^ Registries can also inform regulatory decisions and filling evidence gaps after clinical trials such as uncertainties about long-term safety, optimal use in the real-world setting, and (cost-)effectiveness.^6,10-13^ Besides that, prices for orphan drugs are generally high and exceed cost-effectiveness thresholds.^14,15^ Therefore, national organizations responsible for reimbursement and pricing struggle with decision making leading to delayed or hampered access.^16^ Registries can monitor outcomes and cost-effectiveness in the real world after approval and reimbursement decisions, so-called postauthorization evaluation.^10,15^ This may be accompanied by conditional reimbursement schemes and outcome-based managed entry arrangements,^17^ for which a registry can provide the infrastructure.^18^ When multiple treatment options are available, registries can be used for standardized comparison to enable appropriate use, facilitating a lifecycle approach to drug regulation.^19,20^ It is important to note that knowledge gained through registries may help patients and families in shared decision making and guide expectations in treatment counseling.

Efficient patient registries that serve the purposes described above are crucial to optimize the care of RND. The exact roles of such registries are, however, not yet fully defined, and their implementation is still pioneering work for which practical guidance is lacking. In this study, we provide a practical framework to support the creation and implementation of patient registries, focusing on RND, to guide registry holders. The framework addresses common challenges and elaborates on possible approaches for building a data infrastructure with maximum impact on patient care by serving research, drug development, and regulatory and reimbursement decision making.

## Framework Development Process

**Figure 1:**
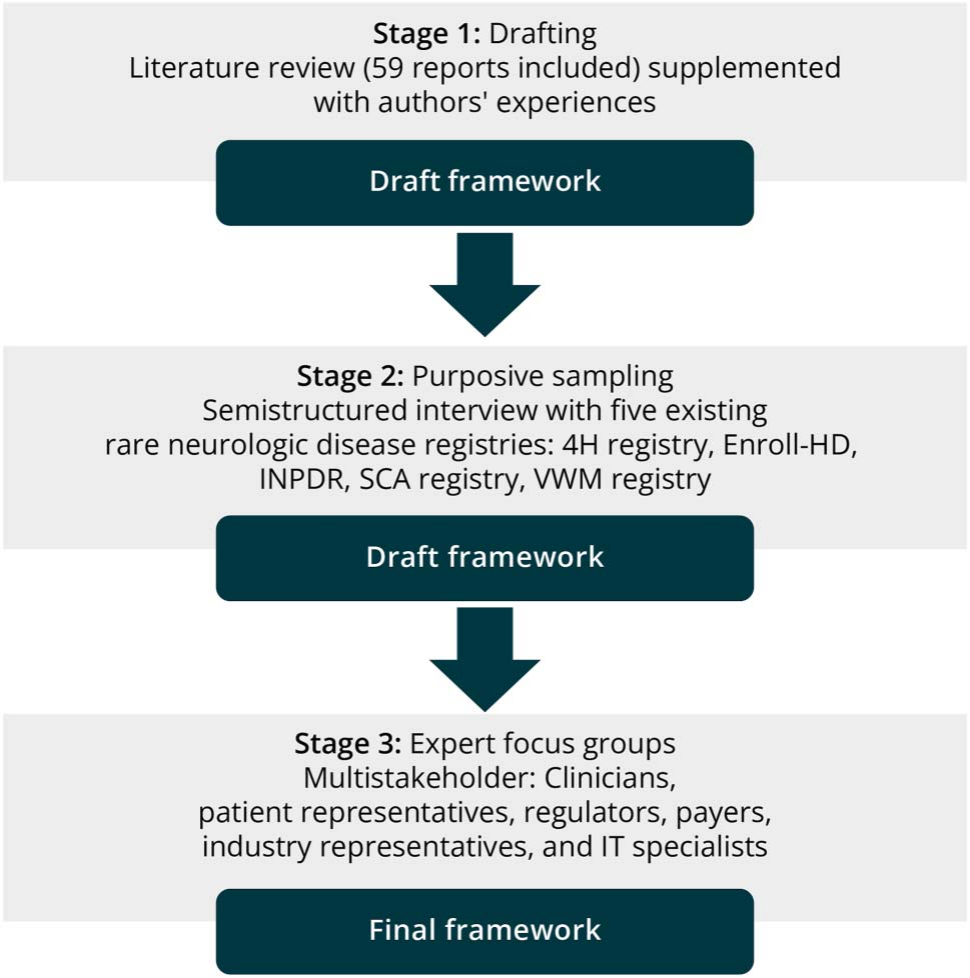
Methodological Flowchart The framework was drafted based on a qualitative literature review and authors' experiences (stage 1), followed by semistructured interviews with existing registries (stage 2). Multistakeholder expert focus groups were done to refine the framework. HD = Huntington disease; INPDR = International Niemann-Pick Disease Registry; IT = information technology; SCA = spinocerebellar ataxias; VWM = Vanishing White Matter.

A group of researchers (D.H.S., C.E.M.H., N.I.W., S.B.) from the MLD initiative^21^ and policy makers (H.P., K.K., L.T., V.V., W.G.) from the Dutch Health Care Institute (health technology assessment [HTA] body), all collaborating in the program “Managing Patient Registries for Expensive Drugs (RORDGM),” initiated this framework. A previous method for a framework about innovating HTA^22^ was adapted to develop this framework. We worked in 3 stages: (1) qualitative systematic literature review to create the draft framework, (2) purposive sampling of 5 established international RND registries using a survey and interviews to collect experiences from registry holders, and (3) expert consultation using multistakeholder focus groups (patient representatives [n = 4], clinicians [n = 6], HTA experts and regulators and payers [n = 7], industry representatives [n = 7], and data/information technology [IT] specialists [n = 5]) to refine the framework (Figure 1). The framework was drafted based on literature (stage 1) to make a comprehensive but manageable overview of the available guidance documents and articles about registries (eAppendix 1 and eFigure 1). The literature was qualitatively analyzed and abstracted using a combined multistep manual deductive and inductive coding approach. In the first step based on the domains used in the program RORDGM (deductive), information on “purpose and data,” “IT infrastructure,” “governance,” and/or “financing” was extracted and ordered. Within these domains, inductive coding in step 2 (ordering, ranking, clustering), step 3 (abstraction, theming, heading), and step 4 (further abstraction, summarizing) led to the draft version of the conceptual framework. The interviews with existing registry holders (stage 2) helped in assessing whether all relevant aspects were addressed and to add practical tips. By using the draft framework as the starting point in stage 3, we were able to structure the discussion with the experts. A detailed description of the steps can be found in eAppendix 2. The framework incorporates standards, that is, best practices, for registry governance, financing, data, and IT infrastructure, categorized into 3 levels: (A) consistent findings from literature, interviews, and focus groups; (B) limited findings from literature, interviews, and focus groups but supplemented and endorsed by the authors; and (C) subject of discussion with inconsistent findings from literature, interviews, or focus groups. The framework also includes tips and tools that may be helpful to fulfil the suggested standards.

### Data Availability

Data not provided in the article because of space limitations, including the list of prioritized topics in the focus groups and the list of participants, may, after approval of those involved, be shared at the request of any qualified investigator for purposes of replicating procedures and results.

## Sample Registries

The sample registries, that is, Enroll-HD (Huntington disease),^23^ International Niemann-Pick Disease Registry (INPDR),^24^ 4H registry (4H leukodystrophy),^25^ SCA registry (spinocerebellar ataxias),^26^ and VWM registry (Vanishing White Matter),^27^ show differences in IT sophistication, data quality, number of employees, and financing models (eTable 1 under eAppendix 3). What they have in common is that emerging treatments are a significant driver of the registries and that the registry purposes are changing over time.

Overall, the interviews highlight the challenges and importance of creating and maintaining rare disease registries, including the need for adequate resources, staff, and IT systems, as well as navigating regulatory standards and involving patients and other stakeholders in the process. Enroll-HD stands out as a highly professional, European Medicines Agency (EMA)–qualified registry. Both Enroll-HD and INPDR praise the clarity and benefits of EMA qualification. INPDR transitioned from physician/researcher-led to community-led, with a trustee board encompassing patient representatives, clinicians, and researchers. The patient-initiated 4H registry outsourced organizational and IT aspects to the Rare-X platform to enable continuation. The academia-driven SCA and VWM registries yielded substantial academic output and accessibility to industry stakeholders, with the SCA registry partly reliant on industry funding.

## Proposed Framework

**Figure 2:**
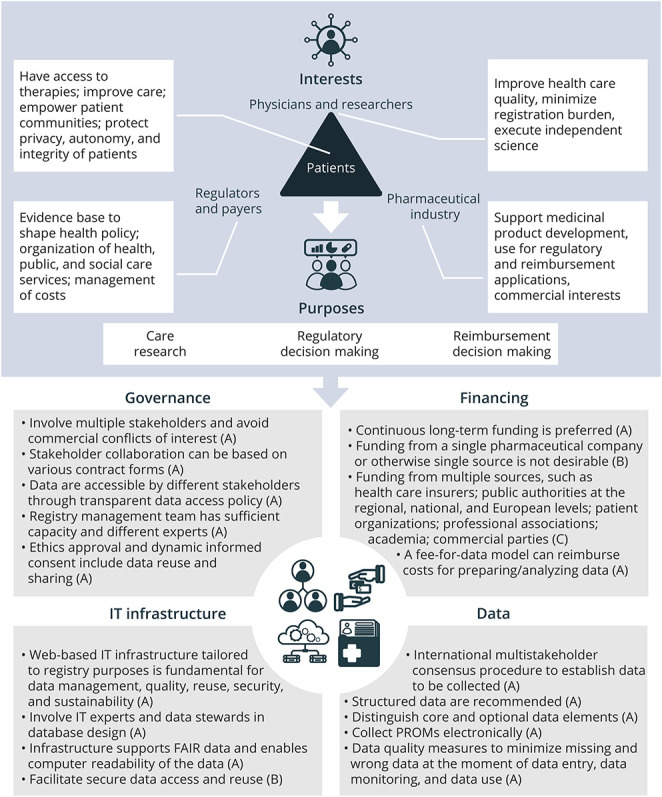
Schematic Summary of the Framework for Rare Disease Registries PROM = patient-reported outcome measure.

The complete framework with the strengths of all recommendations and standards is presented in eTable 2 (under eAppendix 4), with Figure 2 showing a concise visual summary. Key findings and main characteristics are presented in the following.

### Interests and Purposes

#### A Rare Disease Registry Should Be Multipurpose and Serve Different Stakeholders

Stakeholders, including patients, physicians, researchers, regulators, payers, and pharmaceutical industry, have diverse interests in rare disease registries (eTable 3). All stakeholders benefit from high-quality data. Consideration of all interests in registry setup without prioritizing one interest over another is important. The different interests lead to various purposes of rare disease registries including research, care, regulatory, and reimbursement decision making (Table 1). While clear purposes are essential, flexibility and adaptability in registry design are also necessary to accommodate unforeseen future questions. Multipurpose registries are favored over single-purpose ones for rare diseases to avoid scattering, maximizing reuse of scarce rare disease data.

**Table 1:** Data Utilization Purposes

Research
• Prevalence and geographical distribution
• Natural history
• Genotype-phenotype correlations
• Trial execution and design
◦ Addition to trial data
◦ Alternative for placebo (in case this is not ethical)
◦ More effective research and trials
◦ To better reflect the needs of patients in the design of clinical trials
◦ Supporting the setup of (registry-based) studies
• Identifying and developing biomarkers
• Developing outcome measures
• Drug development
• Improving effectiveness
• Appropriate use of drugs
Care
• Supporting health and social service in rare diseases
• Development of disease prevention activities
• Newborn screening programs
• Multiple treatments for a single disease can be compared
• Clinical guideline development
• Shared decision making
• Clinical decision making
• Appropriate use of drug
Regulatory decision making on safety, effectiveness, implementation, and appropriate use
• To gain market approval and access
• Maintaining a marketing authorization
• Long-term monitoring of innovative treatments
• Monitoring effectiveness (periodic reporting of clinical outcomes on individual and aggregated level)
• Pharmacovigilance (expedited and periodic reporting of individual and aggregated adverse events)
• Post-marketing surveillance
• Safety monitoring
Reimbursement decision making
• Effectiveness of treatment
• Providing data for HTA parameters and the appropriate use of drugs
• Comparing treatments and diagnostics
• Identifying subpopulation for treatment
• Mitigating uncertainty in lack of evidence and expensive drugs
• Re-evaluating effectiveness and cost-effectiveness
• Appropriate use arrangements

Abbreviation: HTA = health technology assessment.

### Governance

#### A Transparent Governance Model Should Facilitate Collaboration and (Re)use of Data

A transparent governance model, that allows for some degree of flexibility and is suitable for data utilization, is needed (eTable 2, “Governance > Key governance principles”). Timely data access by various stakeholders should be embedded in the governance model and publicly described to facilitate requests for access by third parties.

#### Broad Registry Ownership to Facilitate Data Use by Third Parties and Ensure Sustainability

There is no consensus on the preferred initiator and owner of registries (eTable 2, “Governance > Owner/initiator”). However, there is consensus that in case of multipurpose registries, broad ownership, for example, multiple academic centers united in an international collaborative network or consortium, with engagement of different stakeholders is recommended. Various stakeholders, such as health authorities, patients, physicians, and pharmaceutical sponsors, are all considered to be suitable registry owners, either individually or in different combinations. A physician-led governance with substantial patient input can be a good option. There is general agreement that registries should not be owned by a single marketing authorization applicant/holder.

Industry-led registries are noted for data quality control and sufficient funding, but conflicts of interest are identified as a downside. Furthermore, pharmaceutical industry might have concerns about data sharing with other companies or stakeholders. Academia-led registries are viewed favorably for their longer term focus and fewer commercial conflicts of interest. Pharmaceutical industry may also prefer registries driven by academic needs, enabling them to align their strategies with the latest research insights. Nonetheless, concerns are expressed about the time-consuming nature of registry management and potential issues regarding sustainable funding and guaranteeing timely data access.

#### Engaging Relevant Stakeholders in Early Registry Development

Collaborative efforts among diverse stakeholders are crucial for a registry's sustained success and impact (eTable 2, “Governance > Stakeholder role and engagement”). Patient advocacy involvement in registry governance is important. These collaborations can be formalized in consortium agreements, in which a specific role for industry, without industry ownership, can be defined. Effective registry implementation requires managing interactions with regulatory authorities and ensuring clear communication among diverse stakeholders.

#### The Registry Management Team Should Include Clinical, Data Management, and Supportive Experts

A proficient and diverse registry management team is essential for day-to-day registry operations, encompassing data processing, quality assurance, and fulfilling of various registry requirements (eTable 2, “Governance > Registry management team”). The team typically includes disease expert clinicians, data stewards, a project leader, and secretarial support. A robust data management strategy with established standard operating procedures is vital. Specialized training for personnel and clear communication channels with a central contact point are necessary.

#### Dynamic Informed Consent for Data Reuse and Negotiating Legal Contracts Take Time

A dynamic and publicly accessible informed consent is deemed essential for data reuse and sharing (eTable 2, “Governance > Ethics, privacy safeguarding and law”). Designing and negotiating consortium agreements and contracts can cause delay in setting up a registry but are essential to regulate collaboration and data reuse by different parties.

### Financing

#### Funding From Multiple Sources to Maintain Independence and Ensure Sustainability

Establishing a sustainable funding model involving different national and international funding sources is critical to ensure long-term sustainability and success in registry endeavors. Potential funders include public institutions, regulatory and reimbursement agencies, academia, and pharmaceutical companies, either individually or in combinations. Conditions for industry funding to maintain independence, integrity, and equitable data access for all stakeholders are added to the framework (eTable 2, “Financing > Sources of funding”).

#### Fees for Data Can Be an Option

Free data sharing is sometimes advocated to endorse therapy development while charging fees may support financial sustainability. Fee structure should take into account the type of requester (e.g., academic vs industry) and the efforts necessary for data delivery.

### Data

#### A Consensus Procedure Involving Multiple Stakeholders to Establish Selection of Data Items

**Figure 3:**
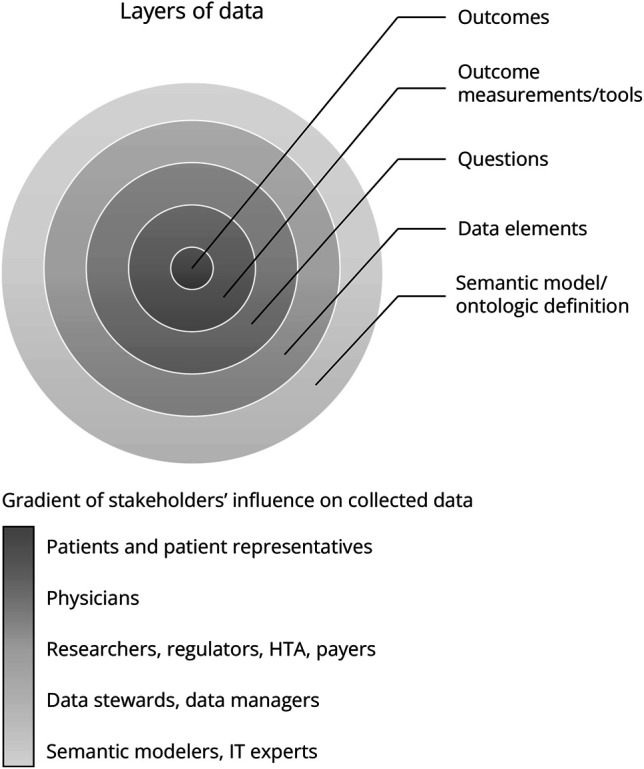
Schematic Visualization of the Gradient of Stakeholders' Influence on Collected Data All stakeholders can be involved in the selection of the collected data in a registry. Their degree of influence depends on the layer of the data. Patients and their advocates should be a central interlocutor in establishing the registry's purposes and data. Physicians with medical and academic expertise can translate patient wishes into relevant outcome measurements/tools and research questions. Regulatory and HTA bodies can formulate research questions and express their data needs. Data stewards and data managers can define data elements for the design of the database. Involving IT specialists at the time of data element establishment is crucial, and for the ontological modelling and computer readability of data elements, input from semantic modelers is needed. HTA = health technology assessment; IT = information technology.

Multipurpose patient registries should involve multiple stakeholders in determining which data are to be collected in the registry (eTable 2, “Data > Procedure to establish data elements”). An international consensus procedure might be an efficient method. Registry contents have different layers, ranging from the meaning of data to the technical coding of data. All these layers should be considered and tailored input from different stakeholders sought, as schematically presented in Figure 3.

#### Distinguish Core and Optional Data Items and Adhere to International Standards

Differentiating core and optional data items is recommended. Implementing standardized core elements in adherence to international standards and ontologies across multiple registries is recommended. Examples are provided in the framework (eTable 2, “Data > Specific data elements”).

#### Use Standardized Tools and Scoring Systems to Collect Neurologic Characteristics

Table 2 presents suggestions for collecting standardized and concise information on important commonalities of RND (Table 2).

**Table 2:** Common Features of Rare Neurogenetic Diseases and Considerations for Data Collection in a Registry

Feature	Considerations
General disease characteristics	
Often progressive	Requires longitudinal follow-up
Interference with neurodevelopment in young children	Structured capturing of developmental motor milestones, for example, according to the WHO motor milestones or the Bayley Scales of Infant and Toddler Development
Variable age at onset	End points that can be applied in wide age ranges
Different disease subtypes	End points and data elements that capture all phenotypes
Genotype-phenotype understanding limited	Collection of genotypes in a registry has a high priority Use HGVS nomenclature
Patient-reported outcomes challenging because of age or cognitive involvement	Proxy-reported versions are mostly available and can be used
Neurologic signs and symptoms	
Gross motor problems	Clinical scoring systems such as the GMFCS, GMFC-MLD, or the mRS are easy to collect in registries and provide crude insights on motor problems Wheelchair dependency might be a valuable binary variable to collect For detailed follow-up, more comprehensive assessments of motor function are necessary
Fine motor problems	Use of clinical scores can be considered, such as the MACS
Speech problems	Use of clinical scores can be considered, such as the CFCS or the ELFC-MLD
Eating and drinking problems	Use of clinical scores can be considered, such as the EDACS
Hearing and vision problems	Part of several standard questionnaires assessing daily functioning, such as the HUI
Cognitive decline	Cognitive screening tools: MMSE or MoCA Neuropsychological assessment, including IQ scores
Psychiatric and behavioral problems	Presence of these problems as binary variable VABSs can be used to assess adaptive behavior (remotely)
Ataxia	Use of clinical scores can be considered, such as SARA, ICARS, and BARS
Spasticity	Presence of these problems as binary variable
Extrapyramidal movement disorders	Videos of movement disorders should be considered, but additional challenges for privacy and mode of storage should be taken into account
Peripheral neuropathy	Presence of these problems as binary variable
Epilepsy	Presence of these problems as binary variable
Urinary and fecal incontinence	Part of several standard questionnaires assessing daily functioning, such as the Katz ADL

Abbreviations: BARS = Brief Ataxia Rating Scale; CFCS = Communication Function Classification System; EDACS = Eating and Drinking Ability Classification System; ELFC-MLD = Expressive Language Function Classification in Metachromatic Leukodystrophy; GMFC-MLD = Gross Motor Function Classification in Metachromatic Leukodystrophy; GMFCS = Gross Motor Function Classification System; HGVS = Human Genome Variation Society; HUI = Health Utilities Index; ICARS = International Cooperative Ataxia Rating Scale; Katz ADL = Katz Index of Independence in Activities of Daily Living; MACS = Manual Ability Classification System; MMSE = Mini-Mental State Examination; MoCA = Montreal Cognitive Assessment; mRS = Modified Rankin Scale; SARA = Scale for the Assessment and Rating of Ataxia; VABS = Vineland Adaptive Behaviour Scale; WHO = World Health Organization.

#### Patient-Reported Outcome Measures Are Valuable and Required for HTA

Patient-reported outcome measures (PROMs) should be disease-specific, culturally adapted, validated, and electronically captured. Especially HTA agencies have considerable interest in PROMs on quality of life, although there is no consensus on the specific tool to be used. Integration of PROMs into standard care during hospital visits helps prospective collection of such data to inform future cost-effectiveness and regulatory questions.

#### Robust Data Quality Measures Are Crucial for the Usability of the Registry

Robust data quality procedures are vital throughout the data lifecycle, including entry, monitoring, and use stages. Strategies included minimizing missing and erroneous data during entry through software tools, varied data sources, and validation. The procedure to obtain an EMA qualification improves data quality. Data quality measures should be tailored to registry objectives to avoid excessive workloads. Frequent updates, longitudinal data, timely data entry after patient visits, and diagnosis checks can enhance data quality. Patient involvement is possible to add missing and update personal data, although concerns exist about the heightened monitoring demands required for patient-entered data because of quality requirements for research data and patients' health literacy.

### IT Infrastructure

#### The IT Infrastructure Should Be Tailored to Registry Purposes and Facilitate Data Management and Data Use

A registry's IT infrastructure is fundamental and supports data management, quality, reuse, analysis, privacy, security, and sustainability. Involving IT experts and data stewards in database design, even with existing software solutions, is important. Flexibility of the system and adherence to findable, accessible, interoperable, and reusable (FAIR) data principles are essential. Customized IT infrastructures developed by professional software developers should be open-source, modular, and secure. Off-the-shelf IT systems are usually accessible and affordable. Multinational registries pose challenges due to varying hospital IT systems, but automated data entry is deemed important for registry success.

Besides capturing or collecting data, the IT infrastructure should facilitate data use. Data sharing or access can be arranged in multiple ways as listed in the framework (eTable 2, “IT infrastructure > Data access”). More advanced registry systems can function as a data analysis framework in which data can be queried instead of just captured.

#### FAIR Data Principles to Maximize Impact and Data Quality

The incorporation of FAIR data principles aids in augmenting data impact and quality. Key terms in the context of FAIR are explained in eAppendix 5, and an example of an ontological data model is presented in eFigure 2. Interoperability and alignment with other registries enable combining different data sources. Although a federated data model is considered beneficial, its current feasibility for a small registry is questioned. Clear perspectives on the required level of FAIR compliance for RND registries are still lacking. Moreover, there are challenges associated with implementing FAIR principles, such as limited availability of FAIR experts and labor-intensive implementation.

## Further Considerations and Discussion

### Framework for Rare Disease Registries

In summary, patient registries for RND may serve different purposes for patients, physicians, researchers, regulators, HTA agencies, payers, and pharmaceutical industry. Despite diverse expectations, aligning registry requirements to accommodate all stakeholders is possible and preferred over multiple registry silos for a single rare disease. This study established standards for rare disease registries through a literature review and qualitative multistakeholder analysis. This framework guides registry holders in optimizing data utilization to accelerate therapeutic access and improve care in rare diseases. The framework addresses purposes for data utilization, governance, financing, data, and IT infrastructure. Key principles include that data should be quickly accessible, independent, and trustworthy. The governance should involve multiple stakeholders, including patient advocates. Data should be highly descriptive, machine-readable, and accessible through a shared infrastructure. Sustainable and independent financing of registries remains difficult.

### Balancing Objectives and Flexibility

The need to define clear objectives at initiation of a registry and at the same time retain flexibility to adapt to future needs is challenging. Clear objectives help to keep focus in a research plan, to limit the number of data elements and associated workload, and to acquire funding for a well-defined project. A possible solution to maintain the registry's flexibility and sustainability lies in its governance model and IT infrastructure. Engaging different stakeholders while maintaining independency anticipates future needs. Independence without commercial interests will ensure that different companies and governmental bodies can trust data from the registry and leverage compliance to commitments such as regulatory postauthorization surveillance.^28^ Legally binding contracts can prevent data loss if ownership changes. An IT infrastructure adhering to FAIR principles facilitates reuse, which again benefits the flexibility of the registry. Striking a balance between clear objectives at the start of the registry and retaining flexibility is essential for accommodating evolving needs in the field.

### Challenges in Real-World Data Acceptance and Quality Assessment

Patient registries collecting real-world data, though valued,^29,30^ face hesitancy in their use. Multiple clinicians, for example, emphasized in the focus groups that natural history studies may offer more rigor and detail but they require more resources and impose higher patient burden and potential biases, for example, regarding participating patients. Multiple stakeholders raised their concerns that payers, regulators, and even journals may be reluctant to trust real-world data, often citing doubts about data quality. Despite evidence showing their noninferiority to more controlled sources such as postapproval trials,^31^ transparent quality standards for registries are lacking. Both registry owners and potential users, including regulatory and HTA authorities, are struggling with this, despite several published guidance documents.^10,32-35^ Currently, the EMA assesses the usability and quality of a registry case-by-case, for example, in a qualification or scientific advice procedure. HTA bodies in Europe and Canada have used EUnetHTA's ReQUEST tool, which aids in creating a comprehensive overview of a registry.^34^ The precise implementation, for example, when regulatory/HTA bodies should complete the tool or when an auto-assessment is helpful, still needs to be defined. In the focus groups, it was also suggested that the European Reference Networks might be involved in rare disease registry assessment in Europe, similar to their auditing activities for expert centers. Specific quality standards or a quality mark, feasible also for small (ultra)rare disease registries, may be helpful.

### International Data for Local Questions

Another complicating factor for use of registries by decision makers noted in the focus groups was that cross-country and even intracountry differences in treatment access and use hamper comparative assessments. In addition, national regulatory evaluations and HTA may lead to local questions for which authorities typically prefer local registries. In rare diseases, adequate powering of these local studies is challenging and often impossible. National regulatory/HTA questions should preferably be accommodated using international registries. The rationale for this becomes even stronger with the EU HTA Regulation being launched in 2025 and enabling joint assessments of advanced therapy medicinal products in Europe. Based on our findings, we suggest that authorities should be open to use international registry data for local questions, which will lead to better and quicker answers than relying only on a small national data pool.

### Perspectives on PROMs

Health-related quality-of-life assessments using PROMs are generally recommended to collect in registries. However, opinions on relevance and purposes vary widely. The focus groups clarified that PROMs are useful in the context of health economic evaluation. For health economics, it was deemed desirable to use the same tool across multiple diseases. On the contrary, disease-tailored PROMs were deemed to be more clinically relevant. Further research is needed on the meaning and the correct application of PROMs in different diseases. The current work of EMA on PROMs and patient experience data in regulatory context is a notable effort.^36^

### Toward Machine-Readable Data

The FAIR data movement promotes machine-readable federated data models.^37^ In practice, this is not always feasible for small registries for (ultra)rare disorders.^20^ There is a gap between technical experts and regulatory/HTA representatives clearly advocating FAIR principles and registry holders, as illustrated in the focus groups where FAIR principles and IT infrastructure were among the least prioritized topics in the focus groups. Practical guidance and registry-specific advice on FAIR principles, especially in lay language, are lacking. Policy makers emphasize the urgency of applying FAIR principles in health data as illustrated by the DARWIN-EU program^38^ and the European Health Data Space. Initiatives such as the personal health train or other federated data analytics systems are promising to overcome scattered health data sets.^39,40^ In rare diseases, there are additional challenges that should be taken into account. For example, the significant amount of unstructured (source) data, resulting from unique disease characteristics and highly specific measurements, is problematic. In addition, for some (ultra)rare diseases, the total number of patients worldwide is so small that a central database is feasible (and maybe preferable) and making various databases machine-actionable not worth the effort. However, maintaining a central database in the long term is labor-intensive and challenging to sustain. Clear definitions for clinical characteristics do not yet exist for many rare diseases, let alone clear data definitions described in semantic ontologies. Still, there was consensus that registry design should involve both physicians and semantic data/IT experts.

### Study Limitations and Future Considerations

This framework for registries was carefully established with input from literature, established registries, and multiple other stakeholder groups. The findings may not be perfectly representative because all groups were represented by a limited number of individuals. In addition, all results should be viewed in light of the present context and may require future updates. Certain topics, such as the collection of genomic data, deserve more attention. Despite these limitations, this framework will benefit registries. Adequate registries are urgent to support decision making about the growing number of emerging new treatments. These treatments, while promising, come with high costs and uncertainties about their long-term effectiveness, posing new research and regulatory challenges. RND registries can play a pivotal role in drug development and evidence generation. Independent and multipurpose patient registries should serve academic research, drug development, regulatory approval, and health care decision making. The proposed framework may guide registry holders, particularly academics and patient advocacy groups, in establishing new and improving existing registries.

## Appendix Authors

**Table:**

Name	Location	Contribution
Daphne H. Schoenmakers, MD	Department of Child Neurology, Emma's Children's Hospital, Amsterdam UMC location Vrije Universiteit; Amsterdam Leukodystrophy Center, Amsterdam Neuroscience, Cellular & Molecular Mechanisms; Medicine for Society, Platform at Amsterdam UMC location University of Amsterdam, the Netherlands	Drafting/revision of the manuscript for content, including medical writing for content; major role in the acquisition of data; study concept or design; analysis or interpretation of data
Sibren van den Berg, MSc	Medicine for Society, Platform at Amsterdam UMC location University of Amsterdam; Department of Endocrinology and Metabolism, Amsterdam UMC location University of Amsterdam, the Netherlands	Drafting/revision of the manuscript for content, including medical writing for content; major role in the acquisition of data; study concept or design; analysis or interpretation of data
Lonneke Timmers, PharmD, PhD	National Health Care Institute (Zorginstituut Nederland), Diemen, the Netherlands	Drafting/revision of the manuscript for content, including medical writing for content; study concept or design
Laura A. Adang, MD, PhD	Division of Child Neurology, Children's Hospital of Philadelphia, PA	Drafting/revision of the manuscript for content, including medical writing for content
Tobias Bäumer, MD	Institute of Systems Motor Science, CBBM, Universität of Lübeck; Centre of Rare Diseases, University Hospital Schleswig Holstein, Lübeck, Germany	Drafting/revision of the manuscript for content, including medical writing for content
Annet Bosch, MD, PhD	Department of Endocrinology and Metabolism, and Division of Metabolic Diseases, Department of Pediatrics, Emma Childrens' Hospital, Amsterdam UMC location University of Amsterdam, the Netherlands	Drafting/revision of the manuscript for content, including medical writing for content
Marc van de Casteele, MD, PhD	National Health Care Institute RIZIV-INAMI, Brussels, Belgium	Drafting/revision of the manuscript for content, including medical writing for content
Mareen R. Datema, PhD	Department of Endocrinology and Metabolism, Amsterdam UMC location University of Amsterdam, the Netherlands	Drafting/revision of the manuscript for content, including medical writing for content
Hanka Dekker, BSc	VKS, Dutch Patient Organization for Metabolic Diseases, Zwolle; United for Metabolic Diseases (UMD), Amsterdam, the Netherlands	Drafting/revision of the manuscript for content, including medical writing for content
Conan Donnelly, PhD	International Niemann-Pick Disease Registry, Washington, Tyne & Wear, United Kingdom	Drafting/revision of the manuscript for content, including medical writing for content
Mariëtte H.E. Driessens, PhD	VSOP-Patient Alliance for Rare and Genetic Diseases, Soest, the Netherlands	Drafting/revision of the manuscript for content, including medical writing for content
Holm Graessner, PhD	Institute for Medical Genetics and Applied Genomics, University of Tübingen; Centre for Rare Disease, University Hospital Tübingen, Germany	Drafting/revision of the manuscript for content, including medical writing for content
Valerie Greger, PhD	Yaya foundation for 4H Leukodystrophy, Minneapolis, MN	Drafting/revision of the manuscript for content, including medical writing for content
Tala Haddad, MSc	Orphanet, INSERM US14 Rare Disease Platform, Paris, France	Drafting/revision of the manuscript for content, including medical writing for content
Günter U. Höglinger, MD	Department of Neurology, LMU University Hospital, Ludwig-Maximilians-Universität (LMU), Munich; German Center for Neurodegenerative Diseases e.V. (DZNE), Munich; Munich Cluster for Systems Neurology (SyNergy), Germany	Drafting/revision of the manuscript for content, including medical writing for content
Hannerieke van den Hout, MD, PhD	Department of Pediatrics, Center for Lysosomal and Metabolic Diseases, Erasmus MC University Medical Center, Sophia Children's Hospital, Rotterdam, the Netherlands	Drafting/revision of the manuscript for content, including medical writing for content
Carla Jonker, PhD	European Medicines Agency, Amsterdam; Medicines Evaluation Board, Utrecht, the Netherlands	Drafting/revision of the manuscript for content, including medical writing for content
Mirjam Langeveld, MD, PhD	Department of Endocrinology and Metabolism, Amsterdam UMC, Amsterdam Gastroenterology Endocrinology Metabolism (AGEM) Research Institute, University of Amsterdam, the Netherlands	Drafting/revision of the manuscript for content, including medical writing for content
Laurie J. Lambert, PhD	Canadian Agency for Drugs and Health Technology Technologies Agendcy in Health (CADTH), Ottawa, Ontario, Canada	Drafting/revision of the manuscript for content, including medical writing for content
Eileen Neacy, MBA	CHDI Management, Inc., the company that manages the scientific activities of CHDI Foundation, Inc., New York, NY	Drafting/revision of the manuscript for content, including medical writing for content
Marc Nieuwland	National Health Care Institute, Diemen, the Netherlands	Drafting/revision of the manuscript for content, including medical writing for content
Thomas Klockgether, MD	German Center for Neurodegenerative Diseases e.V. (DZNE), Munich; Department of Neurology, University of Bonn, Germany	Drafting/revision of the manuscript for content, including medical writing for content
Marjo S. van der Knaap, MD, PhD	Department of Child Neurology, Emma's Children's Hospital, Amsterdam UMC location Vrije Universiteit; Amsterdam Leukodystrophy Center, Amsterdam Neuroscience, Cellular & Molecular Mechanisms; Department of Integrative Neurophysiology, Center for Neurogenomics and Cognitive Research, Vrije Universiteit, Amsterdam, the Netherlands	Drafting/revision of the manuscript for content, including medical writing for content
Andri Papadopoulou, PhD	European Commission, Joint Research Centre (JRC), Ispra, Italy	Drafting/revision of the manuscript for content, including medical writing for content
Kelly Plueschke, PharmD	European Medicines Agency, Amsterdam, the Netherlands	Drafting/revision of the manuscript for content, including medical writing for content
Sanne van Rijn, MSc	Patient Advocate Organization ‘Vereniging HCHWA-d’ (HCHWA-D Association), the Netherlands	Drafting/revision of the manuscript for content, including medical writing for content
Noa Rosenberg, MSc	Medicine for Society, Platform at Amsterdam UMC location University of Amsterdam; Department of Endocrinology and Metabolism, Amsterdam UMC location University of Amsterdam, the Netherlands	Drafting/revision of the manuscript for content, including medical writing for content
Elise F. Saunier-Vivar, PhD	European Leukodystrophies Association, Paris, France	Drafting/revision of the manuscript for content, including medical writing for content
Bruna dos Santos Vieira, MSc	Medical BioSciences Department, Radboud University Medical Center, Nijmegen, the Netherlands	Drafting/revision of the manuscript for content, including medical writing for content
Carla E.M. Hollak, MD, PhD	Medicine for Society, Platform at Amsterdam UMC location University of Amsterdam; Department of Endocrinology and Metabolism, Amsterdam UMC location University of Amsterdam, the Netherlands	Drafting/revision of the manuscript for content, including medical writing for content; study concept or design
Wim G. Goettsch, PhD	National Health Care Institute, Diemen, the Netherlands; WHO Collaborating Centre for Pharmaceutical Policy and Regulation, Division of Pharmacoepidemiology and Clinical Pharmacology, Utrecht University, the Netherlands	Drafting/revision of the manuscript for content, including medical writing for content; study concept or design
Nicole I. Wolf, MD, PhD	Department of Child Neurology, Emma's Children's Hospital, Amsterdam UMC location Vrije Universiteit; Amsterdam Leukodystrophy Center, Amsterdam Neuroscience, Cellular & Molecular Mechanisms, the Netherlands	Drafting/revision of the manuscript for content, including medical writing for content; study concept or design; analysis or interpretation of data

## Glossary

EMA: European Medicines Agency
HD: Huntington disease
HTA: health technology assessment
INPDR: International Niemann-Pick Disease Registry
IT: information technology
PROM: patient-reported outcome measure
RND: rare neurogenetic disease
RORDGM: Managing Patient Registries for Expensive Drugs
SCA: spinocerebellar ataxias
VWM: Vanishing White Matter
